# Hyaline fibromatosis syndrome with mutation c.1074delT of the *CMG2* gene: a case report

**DOI:** 10.1186/1752-1947-8-291

**Published:** 2014-09-03

**Authors:** Imane Cherkaoui Jaouad, Soukaina Guaoua, Aicha Hajjioui, Abdelaziz Sefiani

**Affiliations:** 1 Centre de Génomique Humaine, Faculté de Médecine et de Pharmacie, Université Mohammed V Souissi, Av. Mohamed Belarbi El Alaoui, Rabat 6203 Rabat, Morocco; 2 Département de Génétique Médicale, Institut National d'Hygiène, Av. Ibn Batouta, BP 769, CP 11400 Rabat, Morocco; 3 Centre de pédiatrie, 21, av. 10 Mai, Tétouan, Morocco

**Keywords:** CMG2, Infantile systemic hyalinosis, Hyaline fibromatosis syndrome

## Abstract

**Introduction:**

Juvenile hyaline fibromatosis and infantile systemic hyalinosis are variants of the same autosomal recessive syndrome; hyaline fibromatosis syndrome, characterized by papulonodular skin lesions, gingival hypertrophy, flexion contractures of joints, osteolytic bone lesions and stunted growth. Infantile systemic hyalinosis is distinguished from juvenile hyaline fibromatosis by its more severe phenotype, which includes hyaline deposits in multiple organs, recurrent infections and death within the first two years of life.

Hyaline fibromatosis syndrome is due to mutations of the gene-encoding capillary morphogenesis protein 2 (CMG2). Cases have been reported in different countries but to the best of our knowledge, this is the first reported Moroccan patient with hyaline fibromatosis syndrome and carrying the *CMG2* mutation.

**Case presentation:**

We report the case of an eight-year-old Moroccan male patient with typical features of hyaline fibromatosis syndrome: multiple recurring subcutaneous tumors, gingival hypertrophy, joint contractures and other anomalies carrying a homozygous mutation in the *CMG2* gene. The identification of the mutation in our patient allowed us to do a presymptomatic diagnosis in our patient’s sister, a two-day-old newborn, who is carrying the familial mutation in the heterozygous state. Early recognition of this condition is important for genetic counseling and early treatment.

**Conclusions:**

Hyaline fibromatosis syndrome might be underdiagnosed. Molecular diagnosis will help clinicians and geneticists, firstly to conduct genetic counseling, prenatal diagnosis and early treatment, and secondly to gain better understanding of the disease and genotype-phenotype correlations.

## Introduction

Juvenile hyaline fibromatosis (JHF MIM 228600) and infantile systemic hyalinosis (ISH MIM 236490) are autosomal recessive conditions characterized by multiple subcutaneous skin nodules, gingival hypertrophy, joint contractures and hyaline deposition. The onset of clinical manifestations of JHF is normally in the first three to four months of life, and ISH typically manifests in the first weeks to months of life. In both disorders, mental development is normal.

In JHF, lesions may be nodular and/or papular skin lesions (on the face, neck and especially on retroauricular and perinasal regions), large tumors (especially on the scalp, trunk and limbs) and perianal plaques or nodules. Gingival hypertrophy is common, impairing eating. Bone manifestations include osteolytic lesions (especially in the distal phalanges and metaphyses), cortical thinning and generalized osteopenia. There are reports of scoliosis, macrocephaly and reduced weight and height. The diagnosis is confirmed by the demonstration of hyaline deposition in the dermis. The origin and nature of the amorphous hyaline material have been unclear, but it appears to be principally composed of glycoproteins and glycosaminoglycans [1].

In ISH, in addition to cutaneous lesions, there is articular contracture, gingival hypertrophy and bone abnormalities, and the skin is thick, with hyperpigmentation over bone prominences. The disease progresses with persistent diarrhea, recurrent infections and death within the first two years of life. This disorder shares many similarities with JHF. Clinical presentation is usually at birth or within the first few months, with painful, swollen joint contractures and livid red hyperpigmentation over bony prominences. Histologically, ISH is also characterized by hyaline deposition, but this is more widespread than in JHF and can affect many tissues including skin, skeletal muscle, cardiac muscle, gastrointestinal tract, lymph nodes, spleen, thyroid and adrenal glands [2].

JHF and ISH are two variants of the same disease, now jointly called hyaline fibromatosis syndrome (HFS), this term was first introduced by Nofal *et al*. [3-5]. The first medical case of HFS was reported in 1903 [6]. The disease was first called molluscum fibrosum, then juvenile hyaline fibromatosis and infantile systemic hyalinosis [7], and since it was realized that these were different manifestations of the same disease, the unifying term hyaline fibromatosis syndrome has been adopted [5]. Some 150 medical cases have been reported, showing no geographical or ethnic predisposition. HFS is due to mutations of the gene-encoding capillary morphogenesis protein 2 (*CMG2*) at 4q21. CMG2 is a transmembrane protein that is induced during capillary morphogenesis and that binds laminin and collagen IV via a von Willebrand factor type A (vWA) domain.

Here we describe the first Moroccan HFS patient with typical features characteristic of the syndrome. This patient carries a homozygous mutation of the *CMG2* gene.

## Case presentation

**Figure 1:**
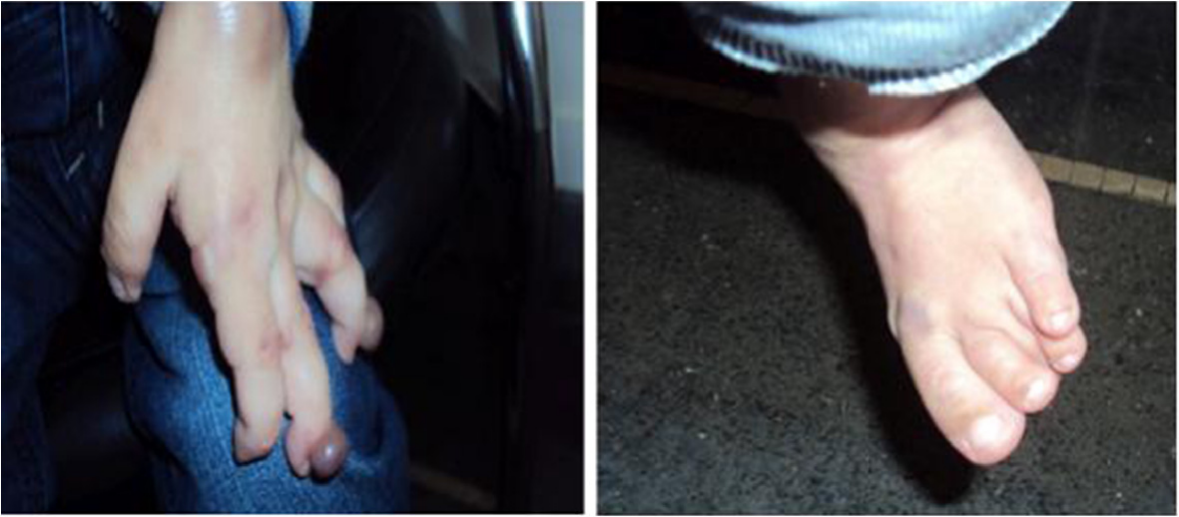
Papular and nodular skin lesions, hyperpigmentation and joint contractures.

An eight-year-old Moroccan male patient was referred to our center for a medical genetics consultation because of the associated presentation of a dysmorphic facies and multiple malformations. He was born to a healthy consanguineous couple, a 27-year-old mother and 40-year-old father. There was a family history of one sister dying at the age of three years with the same symptoms as her brother without a clear diagnosis. The pregnancy was not medically followed, but it was reported to be without complications. At birth, he weighed 2800g, he had a length of 52cm and a head circumference of 36cm. During the first month of life, he appeared to progress normally in his development. A clinical examination at eight-years-old showed a delay in growth development with a weight of 15kg (–3 standard deviation), height of 80cm (–4 standard deviation) and an Occipital Frontal Circumference of 52cm (mean). He presented with joint contractures, painful diffusely thickened skin, hyperpigmentation over joints, papular and/or nodular skin lesions and gingival hypertrophy (Figure 1). His cognitive development was normal. His skin biopsy showed hyaline material accumulation in the dermis.

Based on the clinical pictures and the histological test, a diagnosis of hyaline fibromatosis syndrome grade 2 (HFS) was made. The child was managed conservatively with supportive care given with pain relief, physiotherapy, antimicrobial therapy and dietary modifications.

Informed consent was obtained from the child’s parents prior to implementation of the molecular assay. Peripheral blood was collected from the affected child and his parents.

DNA was extracted from whole blood by conventional methods. Because there is a known hotspot region in exon 13, we amplified this exon using the following primers: CMG2-13F: GCAAGCTTCAGTGAGGGACT and CMG2-13R: GCATGGTATCTGCATTTGGA.

**Figure 2:**
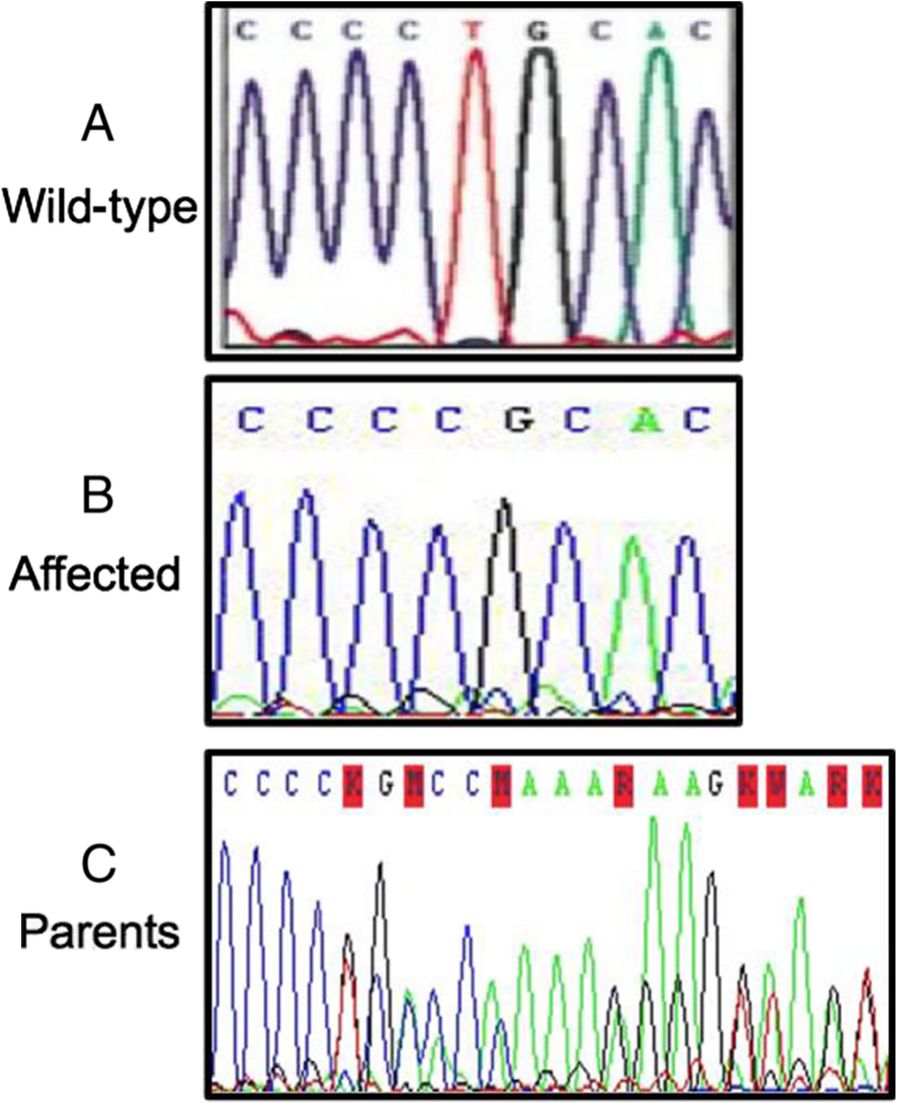
**Electrophoregrams from family with ISH showing wild-type (A) and mutant *CMG2 *sequence (B, C).** HFS, Hyaline fibromatosis syndrome.

Sequencing of the exon 13 of the *CMG2* gene revealed a homozygous deletion in the exon 13 (c.1074delT; p.A359HfsX50; Figure 2B), confirming the diagnosis of HFS. The parents were extensively counseled regarding the diagnosis and its grave outcome.We searched for this mutation in the child’s sister, a two-day-old newborn, who was found to be carrying the familial mutation in the heterozygous state (Figure 2C).

## Discussion

Our case report describes a child who presented with the typical clinical and anatomopathological features of HFS with a progressive and disabling joint pain and contractures.

HFS was recognized as an entity after the first description by Landing and Nadorra in 1986 [8]. This disorder is rare, but it has been recognized in families of various ethnic backgrounds on multiple continents [9]. One report described multiple cases in the Arab population [10], reflecting the presence of consanguinity. HFS is an autosomal recessive disorder that results from mutations in the anthrax toxin receptor 2 gene, ANTXR2 [10]. The gene ANTXR2 *or CMG2* encodes a type I membrane protein that is expressed ubiquitously in the human body [11], with the notable exception of the brain. CMG2 is a type I membrane protein involved in the homeostasis of the extra cellular matrix. This gene was identified as the second receptor for the anthrax toxin. It was found to be responsible for anthrax infection [12] as well as for HFS [13].

The exact cellular role of the protein CMG2 is poorly understood. The best-characterized role of CMG2 is, however, to be the receptor for the anthrax toxin [14,15]. CMG2 enables the anthrax toxin to bind to cells, be internalized and reach the cytosol where it exerts its toxic function. However, several observations have discussed a role in angiogenesis. *CMG2* was originally identified as a gene upregulated in endothelial cells, which play a role in the three-dimensional collagen matrices [12]. In mice, CMG2 was found to be expressed in endothelial cells in the lung, intestine, skin, on smooth muscle cells and in the vascular endothelium [15].

Anthrax toxin receptor 2 is a protein of 488 amino acids which harbors an extracellular von Willebrand A domain (vWA), followed by an uncharacterized immunoglobulin-like domain [15], a transmembrane domain and finally a 148 residue cytosolic tail [14]. The vWA was shown to bind collagen IV and laminin [12]. It contains a metal ion-dependent adhesion site motif with which they interact with extracellular matrix proteins such as collagens. The second extracellular domain of CMG2 is predicted to have an immunoglobulin (Ig) fold [14,15]. Little is known about the transmembrane domain of CMG2, and no structural studies are currently available for the cytosolic tail of CMG2. However, it has been shown that the Ig-like domain contains two disulfide bonds essential for proper ANTXR2 folding in the endoplasmic reticulum [15], and the cytosolic tail plays a role in the interaction with multiple partners during endocytosis. It also contains multiple posttranslational modification sites such as palmitoylation [15], phosphorylation [16] and ubiquitination [16].

More than 150 cases of HFS have been reported and some 34 different mutations, from exon 1 to exon 15, have been identified [14]. Among them, exon 13 is a hotspot for frameshift mutations, which include insertion of one or two bases (c.1073-1074insC and c.1073-1074insCC) and deletion of one base (c.1074delT). Yan *et al*. have shown that these three frameshift mutations represent approximately 60% of all the pathogenic alleles [17]. The frequency of insertions and deletions at positions 1073 to 1074 is likely due to its proximity to a low complexity, GC-rich, region encoding a stretch of proline residues that could constitute a vulnerable site for errors during DNA replication.

Little is known about the consequences of mutations in the *CMG2* gene and especially in exon 13. The frameshift mutation c.1074delT leads to a premature stop codon. The mutated *delT* gene encodes for the ANTXR2 protein has modified and shorter cytosolic tails of 66 amino acids compared with the 148 residue wild-type ANTXR2 tail [17]. Indeed, not only is the site of a mutation is crucial but the nature of the insertion and/or deletion, for example the change in the reading frame.

Therefore, it is important to perform genotype-phenotype correlation at the mRNA, protein, and functional levels, and to analyze the molecular consequences of the specific mutations in exon 13 of the *CMG2* gene in order to evaluate the potential therapeutic targets. Deuquet *et al*. showed that the frameshift mutations in exon 13 lead to a decrease in the mRNA levels of *CMG2*, presumably due to degradation via the nonsense-mediated mRNA decay pathway [14]. Yan *et al.* have shown that c.1073-1074insCC and c.1074delT mutations lead to defects not only at the mRNA level but also at the protein-folding level, limiting the hope for a chemical-based therapy to rescue ANTXR2 expression levels, leaving gene replacement as the only treatment possibility in these patients [17].

Our patient is carrying the mutation c.1074delT in the homozygous state; this single nucleotide deletion in exon 13 was previously reported in patients with different ethnic origins. It was described in a homozygous state in a Kuwaiti family with ISH [3], and in a compound heterozygous state with JHF phenotype in an Italian family [18]. This mutation modifies the open reading frame by a frameshift leading to a change in the cytosolic tail of the protein and a premature stop. This mutation is often associated with a severe form of HFS; a survey of the literature indicates that patients carrying the c.1073-1074insCC or c.1074delT mutations always suffer from the severe form of the disease, while more moderate symptoms are reported for patients with the c.1073-1074insC mutations.

A symptomatic treatment is recommended for HFS. Nonsteroidal anti-inflammatory drugs and opiates help to control pain. When passive movement of joint contractures is painful, physiotherapy should be carried out with care. Early surgical excision of the dermal tumors is indicated for functional and esthetic improvement. Complete excision should be performed in the early phase of tumor development. An intralesional steroid injection may reduce the size of early lesions. A partial gingivectomy may be helpful to treat gingival overgrowth. Oral D-penicillamine has been used in some cases with apparent improvement in joint mobility and flexibility [9].

Cases have been reported from different countries all over the world. To the best of our knowledge, this is the first reported Moroccan patient with this HFS. Here, we describe the clinical and genetic data of a Moroccan patient with HFS. This diagnosis allowed us to provide appropriate management for the patient, to conduct genetic counseling with his family, to do presymptomatic diagnosis and to enrich genetic data on the Moroccan population. We suspect that the prevalence of HFS in Morocco could be high, especially with the high rate of consanguinity in Morocco (15.25%); this leads to an increased level of autosomal recessive diseases [19]. The HFS disease could well be underdiagnosed, supported by the fact that our patient had one sister who passed away in infancy without a precise diagnosis.

## Conclusions

HFS is still a poorly understood disease of a particular biology and raises a problem of treatment. However, the major benefit in identifying pathogenetic mutations in individual cases with HFS is to conduct genetic counseling, prenatal diagnosis and provide early treatment.

## Consent

Written informed consent was obtained from the patient’s legal guardian(s) for publication of this case report and any accompanying images. A copy of the written consent is available for review by the Editor-in-Chief of this journal.

## Abbreviations

ANTXR2: Anthrax toxin receptor 2 gene; CMG2: Capillary morphogenesis protein 2; HFS: Hyaline fibromatosis syndrome; ISH: Infantile systemic hyalinosis; JHF: Juvenile hyaline fibromatosis; vWA: von Willebrand A domain.

## Competing interests

The authors declare that they have no competing interests.

## Authors’ contributions

CJI participated in the conception and design of the study, the sequence alignment and drafted the manuscript. GS participated in the molecular genetic studies and the sequence alignment. HA participated in the clinical diagnosis and the management of the patient. SA conceived of the study, participated in its design and coordination and guided the manuscript draft. All authors read and approved the final manuscript.
